# Microglial Labeling in Mouse Spinal Cord Injury: A Review of Timing, Efficacy, and Specificity

**DOI:** 10.1002/glia.70240

**Published:** 2026-10-19

**Authors:** Ruth Colbert, Julia Di Stefano, Dearbhaile Dooley

**Affiliations:** ^1^ School of Medicine, Health Sciences Centre University College Dublin Dublin 4 Ireland; ^2^ UCD Conway Institute of Biomolecular & Biomedical Research, University College Dublin Dublin 4 Ireland

**Keywords:** antibody markers, microglia, mouse, neuroinflammation, reporter mice, spinal cord injury

## Abstract

Traumatic spinal cord injury (SCI) is a complex and disabling condition with no curative therapy currently available. The mechanical damage of the primary injury phase is followed and exacerbated by a secondary injury phase, characterized by the release of free radicals and pro‐inflammatory cytokines that lead to chronic inflammation. Microglia, as the resident immune cells of the central nervous system, play a critical role in orchestrating this inflammatory response. Studying their dynamics after SCI not only deepens our understanding of injury pathology but also advances the development of microglia‐targeted immunotherapies. However, trauma‐induced transcriptional and phenotypic changes mean that markers used to identify homeostatic microglia may be unreliable when applied to their reactive counterparts. Another major challenge lies within discriminating resident microglia from infiltrating monocyte‐derived macrophages (MDM), which permeate the blood‐spinal cord barrier (BSCB) after injury. In this review, we examine three key variables for selecting appropriate strategies for labeling microglia in mice: (i) time (in relation to injury); (ii) efficacy (within the chosen murine SCI model); and (iii) specificity (to microglia). We discuss the transcriptional landscape of murine microglia pre‐injury and its alteration post‐injury, evaluate the strengths and limitations of commonly used markers, and highlight emerging tools and approaches for studying microglia in preclinical murine SCI research.

## Graphical abstract

- The transcriptional profile of microglia is significantly altered after spinal cord injury (SCI).
- When choosing a marker to label microglia after SCI, it is important to consider its specificity for microglia and its stability of expression.

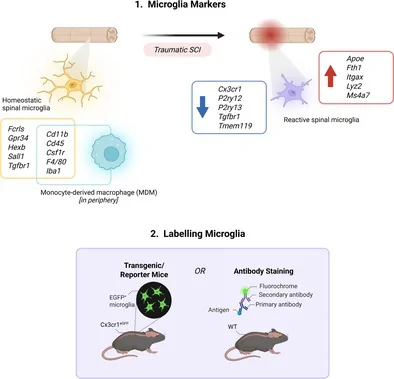

**Abbreviations**
BBB: blood‐brain barrier
BSCB: blood‐spinal cord barrier
CAM: CNS‐associated macrophage
CD: cluster of differentiation
CNS: central nervous system
CPM: cases per million
CSPG: chondroitin sulphate proteoglycan
DAM: disease‐associated microglia
EAE: experimental autoimmune encephalomyelitis
ECFP: enhanced cyan fluorescent protein
EGFP: enhanced green fluorescent protein
FACS: fluorescence‐activated cell sorting
FRed: Fusion Red
IAM: injury‐activated microglia and macrophages
IHC: immunohistochemistry
MACS: magnetic‐activated cell sorting
MDM: monocyte‐derived macrophage
MPS: mononuclear phagocyte system
NGS: next‐generation sequencing
PRR: pattern recognition receptor
R26: Rosa26
RFP: red fluorescent protein
SCI: spinal cord injury
tdT: tdTomato
YFP: yellow fluorescent protein

## 1 Introduction

### 1.1 Pathophysiology of Spinal Cord Injury (SCI)

Traumatic spinal cord injury (SCI) is a complex condition with a high rate of disability and limited treatment options. Its pathophysiology is described in terms of primary and secondary injury phases, the latter of which is further divided into acute (< 48 h), subacute (48 h to 2 weeks), sub‐chronic (2 weeks to 6 months), and chronic (> 6 months) phases (Ahuja et al. 2017). The primary injury phase is defined by damage to the spinal cord directly caused by the physical trauma, which can result from compression, transection, or contusion of the spinal cord (Hachem and Fehlings 2021). The immediate aftermath of this trauma includes damage to the local vasculature, disruption of the blood‐spinal cord barrier (BSCB), neuronal and glial cell death, and demyelination (Ahuja et al. 2017; Hejrati and Fehlings 2021).

These events trigger the secondary injury phase, involving a series of molecular and cellular cascades, which exacerbate the primary injury and persist. In the acute phase of secondary injury, vascular damage and subsequent hypoperfusion of the injured spinal cord lead to ischemia and ultimately, necrosis (Ahuja et al. 2017). The acute phase is further characterized by excess glutamate‐associated excitotoxicity, oxidative stress from free radicals, and induction of apoptotic signaling, which collectively contribute to secondary tissue damage and neuronal and glial cell death (Ahuja et al. 2017; Hachem and Fehlings 2021) within the injured cord. As the injury progresses through the subacute and subchronic phases, axons are subject to Wallerian degeneration (Ahuja et al. 2017). A glial scar is formed around the cystic cavity within the lesion, and this is fundamentally associated with reactive astrocytes and chondroitin sulphate proteoglycans (CSPG), which are inhibitors of axonal regrowth (Ahuja et al. 2017; Hachem and Fehlings 2021; Hejrati and Fehlings 2021; Pang et al. 2021). Additional growth inhibitory signals present in the injury milieu include Nogo‐A and myelin‐associated glycoprotein (Ahuja et al. 2017; Hachem and Fehlings 2021). Altogether, the regeneration potential of the spinal cord, which is already limited in a noninjured state, is diminished in the lesion microenvironment (Ahuja et al. 2017). In parallel, inflammation plays a pivotal role throughout the secondary injury phase of SCI, and this response is largely driven by two immune cell populations: central nervous system (CNS)‐resident microglia and peripheral monocyte‐derived macrophages (MDM) which permeate the damaged BSCB (Hachem and Fehlings 2021). Microglia and MDMs, along with other infiltrating immune cells, create a considerable pro‐inflammatory environment through the release and regulation of cytokines and chemokines (Michel et al. 2021). This inflammatory milieu can exacerbate tissue damage following the initial trauma insult. Despite their detrimental inflammatory roles, microglia and MDMs also play important beneficial functions, including phagocytosis and the clearance of cellular debris during the post‐injury response (David and Kroner 2011). However, the acute and sustained nature of neuroinflammation following SCI can ultimately render this response maladaptive, contributing to secondary tissue damage and impaired recovery (Paolicelli et al. 2022). The secondary inflammatory response can, however, be targeted with immunotherapies and in a recent bibliometric review by Li et al., inflammation was identified as a top research frontier in the field of SCI research (Li, Wei, et al. 2022). As resident immune cells in the spinal cord, research into microglia can provide a clearer understanding of the pathological molecular mechanisms in SCI, and these cells also present a target for immunotherapies.

### 1.2 The Nomenclature of Microglia in SCI

As resident immune cells of the CNS parenchyma (Paolicelli et al. 2022), microglia are commonly referred to as “CNS‐resident macrophages”. In the context of inflammation, microglia and MDMs overlap (although not fully) with respect to function, morphology, and surface marker expression (Ransohoff 2016). However, microglia have a distinct ontogeny (Alliot et al. 1999; Ginhoux et al. 2010; Ng et al. 2023), function within their microenvironment (Paolicelli et al. 2022; Prinz et al. 2019), and transcriptional signature (Butovsky et al. 2014; Gautier et al. 2012; Grassivaro et al. 2020; Hickman et al. 2013). To begin, microglia derive from the embryonic yolk sac (Alliot et al. 1999; Ginhoux et al. 2010; Schulz et al. 2012), and thereafter are maintained by local proliferation (Ajami et al. 2007; Alliot et al. 1999) in the CNS. In contrast, MDMs differentiate from bone marrow‐derived monocytes (Ng et al. 2023). Microglia serve a variety of functions, critical for the developing, adult, and aging CNS. The ability of microglia to engage in surveillance, phagocytosis, and secretion of factors facilitates their role in innate immunity (Paolicelli et al. 2022). However, their contribution as glial cells extends to and includes neurogenesis, vasculogenesis, myelination, and synaptic remodeling (Paolicelli et al. 2022; Prinz et al. 2019).

While in the past microglia have been (and in many cases still are) categorized as “resting” or “activated”, these terms are no longer widely accepted. Even in the healthy CNS, microglia are never resting, but are constantly surveying their environment through motility of their processes (Davalos et al. 2005; Nimmerjahn et al. 2005). In a physiological environment, therefore, microglia are described as “homeostatic,” rather than “resting” (Paolicelli et al. 2022). While microglia are as such always active, in pathology, they become “reactive” to their microenvironment, and this leads to functional, transcriptional, metabolic, and morphological changes (Paolicelli et al. 2022) in these dynamic cells. The concept of “*M1*” and “*M2*” labels to categorize microglia subtypes during inflammation has similarly been revised. These opposing states are synonymous with “inflammatory” (or “classically activated”) and “anti‐inflammatory” (or “alternatively activated”) labels, respectively. However, this *M1/M2* theory arose from experiments with MDMs in vitro (Ransohoff 2016), and was merely extended to microglia given their analogous inflammatory role in the CNS. The advent of single‐cell transcriptomics has demonstrated that reactive microglia do not adopt one of two discrete, opposing states, but instead occupy a continuum of transcriptionally distinct states shaped by disease context, region, and time (Fisher et al. 2022; Masuda et al. 2019). This has led to the identification of context‐specific microglial signatures, such as the disease‐associated microglia (DAM) phenotype first described in a model of Alzheimer's disease (Keren‐Shaul et al. 2017), and in the context of SCI, the injury‐activated microglia and macrophages (IAM) phenotype (Wahane et al. 2021). These findings reinforce that the M1/M2 dichotomy fails to recognize the multidimensional and multivariate states occupied by microglia in a neuroinflammatory environment (Paolicelli et al. 2022), which should be considered when characterizing these immune cells in the context of SCI.

### 1.3 Studying Microglia in SCI

The transcriptional (among others, e.g., metabolomic, proteomic) profile of microglia will change following SCI as microglia respond to the injury environment and adopt different functional states (Fisher et al. 2022; Hakim et al. 2021; Li, Wu, et al. 2022; Milich et al. 2021). While certain genes (often referred to as “markers of activation”) may be upregulated or exclusively expressed in response to injury, many homeostatic markers will be downregulated in injury (and disease; Keren‐Shaul et al. 2017; Masuda et al. 2020; Milich et al. 2021; Wahane et al. 2021). While there are select core genes expressed by microglia alone, many typical microglia markers are also expressed by MDMs as well as CNS‐associated macrophages (CAMs): perivascular, leptomeningeal, and choroid plexus macrophages (Prinz et al. 2019). In the healthy CNS, peripheral MDMs are absent, therefore, shared markers will only label microglia (and potentially CAMs). There is more complexity after SCI, as MDMs (and other peripheral immune cells) cross the compromised BSCB and enter the CNS (Fenrich et al. 2013; Hachem and Fehlings 2021). Knowledge of distinct and context‐appropriate microglial markers across the temporal phases of SCI will strengthen the field and improve the interpretation of experimental findings. Given their potential as therapeutic targets, a deeper understanding of microglial responses to trauma is essential to advance the development of effective immunotherapies for SCI.

Within this review, we focus on the role of microglia as an innate immune cell. Specifically, we examine how microglia can be labeled for study in the context of preclinical murine SCI research. We primarily consider microglial markers with respect to the following variables: (i) *time* (in relation to injury; encompassing pre‐injury, acute, subacute, subchronic, and chronic injury phases); (ii) *efficacy* (in the murine injury model); and (iii) *specificity* (to microglia). Additionally, we summarize the approaches available to study labeled microglia in the mouse, including advances in imaging and transgenic technologies. Within this review, we define “microglia‐specific” as a gene which (i) in the CNS parenchyma, is exclusively expressed by microglia and (ii) in the periphery, is not expressed by monocytes or MDMs.

## 2 Homeostatic Microglial Markers (i.e., Pre‐Injury) and Experimental Approaches to Study Labeled Microglia in the Mouse

### 2.1 Early Microglial Studies Using 2D Microscopy and Flow Cytometry

Microglia were first described by Río‐Hortega in 1919 (Sierra et al. 2016). Within this series of four seminal papers, Río‐Hortega recognized the morphological and functional similarities between this newly discovered type of glial cell and peripheral leukocytes. It was therefore natural that, when immunohistochemistry (IHC) was first applied to visualize microglia (Hume et al. 1983), these early studies utilized antibodies against known macrophage markers, such as F4/80 (Perry et al. 1985), Cd68 (ED‐1 clone; Dijkstra et al. 1985; Holness and Simmons 1993), and Cd11b (OX‐42 clone; Robinson et al. 1986). IHC (with confocal laser scanning or similar 2D microscopy) remains the most common and accessible approach used to study microglia. Both the orphan receptor F4/80 (*Adgre1*/*Emr1*) and the cluster of differentiation (CD) antigen Cd68 are still widely used for immunohistochemical visualization of microglia. Cd11b (*Itgam*) is now typically used in conjunction with Cd45 (*Ptprc*) as the most common gating strategy to isolate microglia using flow cytometry. While both are pan‐macrophage markers, microglia will present as Cd11b^+^Cd45^lo^ while MDMs are Cd11b^+^Cd45^hi^ (Akiyama and McGeer 1990; Garcia et al. 2014; Sedgwick et al. 1991). Both IHC/2D microscopy and flow cytometry can utilize a combination of antibodies, facilitating labeling with marker(s) of choice and simultaneously other genes of interest.

The prototypical antibody marker for microglia is Iba1 (*Aif1*). The discovery of this gene and its expression in microglia was first described (in rat) in 1996 (Imai et al. 1996). Shortly after, IHC confirmed the colocalization of Iba1 with previously established microglial markers (Cd11b, Cd68) (Ito et al. 1998). These two studies demonstrated that in the rodent brain, Iba1 is specifically expressed by microglia as well as perivascular, leptomeningeal and choroid plexus macrophages (i.e., CAMs). However, it was also found to be expressed by MDMs in the periphery. Several other microglial markers were then identified [e.g., *Cx3cr1* (Harrison et al. 1998; Jung et al. 2000), *Csf1r* (Sasmono et al. 2003), *P2ry12* (Sasaki et al. 2003)] in rodents before the use of microarray enabled a more high‐throughput approach. Together with the advancement of next‐generation sequencing (NGS), namely bulk (Mortazavi et al. 2008; Nagalakshmi et al. 2008) and single‐cell (Tang et al. 2009) RNA sequencing (RNA‐seq), we can now describe in detail the transcriptional signature of microglia in both the healthy and diseased/injured murine CNS. As a prerequisite to these transcriptomic approaches, microglia will be sorted using fluorescence‐ or magnetic‐activated cell sorting (FACS or MACS). Like flow cytometry, microglia will typically be gated based on Cd11b^+^Cd45^lo^ expression. Section 3 will describe the transcriptional profile of microglia after SCI in the mouse, which has been elucidated through sequencing studies. Here, we will begin by characterizing the marker expression of healthy murine microglia, that is, pre‐SCI.

### 2.2 Microglial Profiling With Advances in Microarray and Next‐Generation Sequencing (NGS)

Bédard et al. were the first to identify novel microglial genes using gene array in the mouse (Bédard et al. 2007). *F11r*, *Gpr12 (P2ry12)*, *Gpr34*, and *Msr2 (Fcrls)* were identified as being predominantly expressed by microglia; *Gpr84*, *Gpr86 (P2ry13)*, *Olfml3*, *Siglech*, and *Stab1* were expressed by (spleen) macrophages as well as microglia. All markers were confirmed to be colocalized with Iba1 using in situ hybridization. *Stab1* was also expressed in CAMs, while the expression of the other markers was localized in parenchymal microglia. Expression of many known monocytic lineage markers was also compared across microglia, monocytes, and spleen macrophages: *Aif1, Cd68*, *Csf1r*, *Cx3cr1*, *Emr1* (*Adgre1*), *Il10ra*, *Itgam*, *Tgfbr1*, and *Trem2* were among those preferentially or comparably expressed in microglia. *Ptprc* was also detected in microglia but with lower expression. Microglia were subsequently profiled by microarray as part of the Immunological Genome (ImmGen) Project (Gautier et al. 2012). This study revealed the transcriptional diversity among distinct resident tissue macrophages in the mouse, and microglia were found to have low expression of hundreds of genes that were expressed by other macrophages (red pulp splenic, lung, and peritoneal). Microglia genes (defined in this study as having expression greater than five‐fold compared to the other macrophage populations) included *Cx3cr1*, *Fcrls*, *Gpr84*, *Hexb*, *Olfml3*, *Sall1*, *Serpine2*, *Siglech*, *Slc2a5*, *Sparc*, *Tmem119*, and *Trem2*. *Tgfbr1*, *P2ry12*, and *Itgam* were found to be enriched in both microglia and splenic, lung, or peritoneal macrophages, respectively.

Butovsky et al. are attributed with describing the first microglial “signature”, similarly using microarray, as well as mass spectrometry (Butovsky et al. 2014). Genes uniquely or highly expressed in microglia (CD11b^+^CD45^lo^) versus peripheral immune cells were initially selected. The authors particularly focused on monocytes (CD11b^+^Ly6C^+^) among the peripheral immune cells, as known infiltrators of the CNS during neuroinflammation (King et al. 2009). These genes were then filtered against the profiles of other CNS cells (neurons, astrocytes, oligodendrocytes). A microglial signature comprising 106 genes was determined and the top genes enriched in microglia included *C1qa, C1qb, Csf1r, Ctss, Cx3cr1, Gpr34, Hexb, Mafb, Olfml3, P2ry12, P2ry13*, and *Tmem119*; other highly expressed genes included *Fcgr1, Fcrls, Siglech*, and *Tgfbr1*. In comparison, 81 genes were found to be enriched in both microglia and other organ‐specific tissue‐resident macrophages (F4/80^+^CD11b^+^). Grassivaro et al. have compared transcriptomic profiles of microglia (Cd11b^hi^Cd45^lo^) and (liver) macrophages (Cd11b^hi^Cd45^hi^) across development and adulthood in the mouse, using microarray (Grassivaro et al. 2020). Interestingly, the peripheral macrophages were found to express a subset of known microglia‐specific markers during development, (e.g., *Aif, Cx3cr1, Gpr84, Itgam, Socs3*, and *Tyrobp*), although some were at levels much lower than in microglia (e.g., *Fcrls, Hexb, Itgb5, P2ry12, P2ry13, Tmem119*, and *Trem2*). A core 65 transcripts were identified as homeostatic microglial markers conserved across development and adulthood. Among these were 14 markers previously published as being microglia‐specific (in this study, defined as the absence of expression by MDMs): *Crybb1, Garnl3, Gpr34, Lag3, Nuak1, Olfml3, Rtn1, Rtn4rl1, Sall1, Sall3, Siglech, Slc1a3, Sparc*, and *Tnfrsf17*. Embryonic, juvenile, and adult microglia have also been profiled using single‐cell RNA‐seq (Masuda et al. 2019). Healthy postnatal murine microglia highly express genes including *Cst3, Selplg, Slc2a5, Sparc*, and *Tmem119*. Mrdjen et al. profiled all the immune cells within the CNS, through mass and fluorescence cytometry (Mrdjen et al. 2018). In the healthy CNS, microglia are the sole immune cell type residing in the parenchyma, while CAMs and dendritic cells are found within the other compartments (i.e., choroid plexus of the ventricles, meninges, and perivascular space of the blood–brain barrier (BBB) and BSCB). Mrdjen et al. provide a panel of 12 markers to distinguish microglia (Cd45^+^Siglech^+^Cx3cr1^+^) from CAMs (Cd206^+^Cd38^+^Siglec1^+^), monocyte‐derived cells (Ly6c^+^Cd14^+^Cd44^+^), and dendritic cells (Cd11c^+^MHCII^+^Cd43^+^) in the mouse. Applicable in steady state and neuroinflammation, this combination of markers is a useful tool for preclinical microglial research.

Microglia are exceptionally responsive to their local environment, with surveillance being a characteristic behavior of these immune cells. To this end, Hickman et al. have characterized a microglial “sensome”, representing the top 100 genes enriched in microglia (versus all other cell types in the brain) which serve as sensors to changes in their environment (Hickman et al. 2013). Functionally, these genes include pattern recognition receptors (PRRs) (e.g., *Cd68, Clec7a, Selplg, Trem2*), cytokine and chemokine receptors (e.g., *Ccr5, Csf1r, Cx3cr1*, and *Tgfbr1*), ECM receptors (e.g., *Emr1 (Aif1)*, *Itgam*, and *Ptprc*), purinergic receptors (e.g., *Gpr34, P2ry12*, and *P2ry13*), among other receptor classes (e.g., *Gpr84, Siglech, Slc2a5, Tmem119*, and *Tnfrsf17*). Hickman et al. then used direct RNA‐seq to determine the molecular signature of microglia, comparing the microglial transcriptome to that of (peritoneal) macrophages and whole brain in the mouse. Comparing the top 10% of transcripts expressed in microglia (Cd11b^hi^Cd45^lo^) to the top 10% for peripheral macrophages (Cd11b^hi^Cd45^hi^), two‐thirds were expressed by both cell types, with a third of transcripts unique to each. As noted by the authors, this affirms that microglia require a subset of genes specific to their local environment, differing from that of peripheral macrophages. The top genes enriched in microglia included *Cx3cr1, Fcrls, Fos, Gpr34, Hexb, Olfml3, P2ry12, Rnase4, S100a8, S100a9, Siglech*, and *Tmem119*, several of which are sensome genes. Several studies have employed single‐cell RNA‐seq to unravel the transcriptional changes occurring in murine microglia after SCI. In the uninjured spinal cord, microglia comprise the “homeostatic” cluster(s) which have been defined by expression of genes such as *Aif1, Btg2, C1qb, Csf1r, Cst3, Cx3cr1, Egr1, Egr3, Fcrls, Fos, Gpr34, Hexb, Hspa1a, Itgb5, Jun, Klf2, Mertk, Olfml3, P2ry12, P2ry13, Plxdc2, Ptprc, Rnase4, Sall1, Selplg, Serpine2, Siglech, Slc2a5, Sparc, Tgfbr1*, and *Tmem119* (Brennan et al. 2022; Hakim et al. 2021; Hamel et al. 2023; Li, Wu, et al. 2022; Milich et al. 2021; Wahane et al. 2021; Wang et al. 2022). Table 1 lists the microglia‐enriched genes mentioned in‐text with their corresponding protein name and subcellular location.

**TABLE 1:** Microglia‐enriched genes in the mouse described in Section 2 with corresponding protein names and subcellular locations.

Gene	Protein name	Subcellular location
Adgre1 (Emr1)	Adhesion G protein‐coupled receptor E1; EGF‐like module receptor 1; F4/80	Plasma membrane
Aif1 (Iba1)	Allograft inflammatory factor 1; Ionized calcium‐binding adapter molecule 1	Cytoplasm
Anxa3	Annexin A3	Cytoplasm
Bhlhe41	Class E basic helix–loop–helix protein 41	Nucleus
Btg2 (Tis21)	BTG family member 2	Cytoplasm
C1qa	Complement C1q subcomponent subunit A	Secreted
C1qb	Complement C1q subcomponent subunit B	Secreted
Ccr5	C‐C chemokine receptor type 5 (CD195)	Plasma membrane
Cd14	Cluster of differentiation 14 (CD14)	Plasma membrane
Cd68	Macrosialin (CD68)	Plasma membrane
Clec7a	C‐type lectin domain family 7 member A	Plasma membrane
Crybb1	Beta‐crystallin B1	Unknown
Csf1r (c‐fms)	Macrophage colony‐stimulating factor 1 receptor (M‐CSF) (CD115)	Plasma membrane
Cst3	Cystatin C	Secreted
Cts1 (Cts7)	Cathepsin 1; Cathepsin 7	Lysosome/secreted
Ctsd	Cathepsin D	Lysosome/secreted
Ctss	Cathepsin S	Lysosome/secreted
Cx3cr1	CX3C chemokine receptor 1; Fractalkine receptor	Plasma membrane
Egr1	Early growth response protein 1	Nucleus
Egr3	Early growth response protein 3	Nucleus
F11r (Jam1)	Junctional adhesion molecule A (CD321)	Plasma membrane
Fcgr1	High affinity immunoglobulin gamma Fc receptor I (CD64)	Plasma membrane
Fcrl2 (Fcrls)	Fc receptor‐like S, scavenger receptor	Plasma membrane
Fos	Protein c‐Fos	Nucleus
Garnl3	GTPase‐activating Rap/Ran‐GAP domain‐like protein 3	Unknown
Gpr34	Probable G‐protein coupled receptor 34	Plasma membrane
Gpr84	G‐protein coupled receptor 84	Plasma membrane
Hexb	Beta‐hexosaminidase subunit beta	Lysosome
Hspa1a	Heat shock 70 kDa protein 1 (Hsp72)	Cytoplasm
Il10ra	Interleukin‐10 receptor subunit alpha (CD210)	Plasma membrane
Itgam (Cd11b)	Integrin alpha‐M; CD11 antigen‐like family member B (CD11b)	Plasma membrane
Itgb5	Integrin beta‐5	Plasma membrane
Jun	Transcription factor c‐Jun	Nucleus
Klf2	Krüppel‐like factor 2	Nucleus
Lag3	Lymphocyte activation gene 3 protein (CD223)	Plasma membrane
Lgmn	Legumain	Lysosome
Mafb	Transcription factor MafB	Nucleus
Mertk	Tyrosine‐protein kinase Mer	Plasma membrane
Nuak1	NUAK family SNF1‐like kinase 1	Cytoplasm
Olfml3	Olfactomedin‐like protein 3	Secreted
P2ry12	P2Y purinoceptor 12	Plasma membrane
P2ry13	P2Y purinoceptor 13	Plasma membrane
Plxdc2	Plexin domain‐containing protein 2	Plasma membrane
Ptprc (Cd45)	Receptor‐type tyrosine‐protein phosphatase C (CD45)	Plasma membrane
Rnase4	Ribonuclease 4	Secreted
Rtn1	Reticulon‐1	Endoplasmic reticulum/Golgi apparatus membrane
Rtn4rl1	Reticulon‐4 receptor‐like 1	Plasma membrane
S100a8	S100 calcium‐binding protein A8; Calgranulin‐A	Cytoplasm
S100a9	S100 calcium‐binding protein A9; Calgranulin‐B	Cytoplasm
Sall1	Sal‐like protein 1	Nucleus
Sall3	Sal‐like protein 3	Nucleus
Selplg	P‐selectin glycoprotein ligand 1 (CD162)	Plasma membrane
Serpine2	Glia‐derived nexin	Secreted
Siglech	Sialic acid binding Ig‐like lectin H	Plasma membrane
Slc1a3 (Eaat1, Glast1)	Solute carrier family 1 member 3; Excitatory amino acid; Sodium‐dependent glutamate/aspartate transporter 1 transporter 1	Plasma membrane
Slc2a5 (Glut5)	Solute carrier family 2, facilitated glucose transporter member 5; Fructose transporter	Plasma membrane
Sparc	Secreted protein acidic and rich in cysteine; Osteonectin	Unknown
Tgfbr1	Transforming growth factor‐beta receptor type I	Plasma membrane
Tmem119	Transmembrane protein 119	Plasma membrane
Tmsb4x	Thymosin beta‐4	Cytoplasm
Tnfrsf17	Tumor necrosis factor receptor superfamily member 17	Plasma membrane
Trem2	Triggering receptor expressed on myeloid cells 2	Plasma membrane

### 2.3 3D Microscopy Applications for the Study of Spinal Microglia In Vivo

3D microscopy has emerged as a viable approach to study labeled microglia in the mouse, including in SCI. While 2D microscopy (and flow cytometry) can use either antibody‐stained tissue or tissue from transgenic reporter tools, 3D microscopy requires the use of reporter mice to facilitate in vivo imaging. The development of Cx3cr1^GFP^ reporter mice (Jung et al. 2000; evaluated in Section 3) presented the first opportunity for in vivo imaging of microglia, which were leveraged in early studies of intravital imaging of microglia (Davalos et al. 2005; Nimmerjahn et al. 2005). Microglia were initially visualized in the cortex, which is more straightforward than in the spinal cord. A major challenge associated with imaging in the live spinal cord is the introduction of motion artifacts from the animal's heartbeat and breathing, due to the proximity of these organs to the spinal cord. Spinal microglia were first imaged successfully in vivo in mice via the implementation of a customized stabilization device that immobilized the spinal cord and deep anesthesia (Davalos et al. 2008). Similarly, Evans et al. carried out in vivo imaging of microglia in a crush model of SCI (Evans et al. 2014). Evans et al. also leveraged the differential susceptibility of microglia and monocytes to radiation (Matsumoto and Fujiwara 1987), using bone marrow chimeras to distinguish these cells. In this study, the authors found that re‐opening of the spinal column prior to imaging had less of an inflammatory response compared to chronic window implantation. In vivo imaging of microglia in the spinal cord can alternatively be performed by paralyzing the animal and providing artificial ventilation and this has been applied in a laser‐induced model of focal SCI (Nadrigny et al. 2017). 3D imaging can also be performed ex vivo. Noristani et al. have combined tissue clearing and two‐photon microscopy in a transection model of SCI (Noristani, Boukhaddaoui, et al. 2017). This high‐resolution imaging technique enabled a spatiotemporal assessment of microglial cell density and morphology in the lesion.

While Cx3cr1^GFP^ mice are consistently used in the aforementioned studies, Masuda et al. and Ruan et al. have demonstrated that Hexb^tdTomato^ and Tmem119^tdTomato^ reporter mice, respectively, are suitable for intravital two‐photon imaging of microglia via a cranial window (Masuda et al. 2020; Ruan et al. 2020). Both reporter lines were tested in steady‐state and after a laser‐induced focal brain injury. Fenrich et al. have further employed intravital two‐photon imaging in Thy1^CFP^LysM^GFP^CD11c^EYFP^ triple reporter mice to study the interactions between resident and infiltrating myelomonocytic cells and axons in the spinal cord, in a unilateral pin‐prick model of SCI (Fenrich et al. 2013). This was the first study describing the influx of resident and infiltrating myelomonocytic cells in vivo, after SCI. In the injury model, Cd11c^+^ cells were Iba1^+^, and had morphology consistent with reactive microglia.

Chronic in vivo imaging of microglia in the murine spinal cord has also been achieved, using an implanted chamber (Farrar et al. 2012). The use of an implantation facilitates longitudinal imaging studies without the need for repeated surgeries; previous studies repeatedly opened the imaging window for each imaging session (Davalos et al. 2008; Dibaj et al. 2010). When using in vivo imaging to study the inflammatory response in SCI, it is important to consider potential confounding inflammation from the surgical procedure to expose the spinal cord. Farrar et al. reported a mild inflammatory response in microglia, acutely after implantation of the imaging chamber (Farrar et al. 2012). However, this surgery‐associated inflammation could be avoided if the chamber was implanted several weeks before the first imaging session. Wu et al. similarly tested for an inflammatory response after a conventional surgical imaging chamber implant and reported microglial reactivity (Wu et al. 2022). However, Wu et al. go on to describe a protocol for a novel intervertebral window which is minimally invasive and does not induce an inflammatory response. The optical window surgical procedure preserved the animal's ligamentum flavum, which was subject to optical clearing. Its overall design facilitated long‐term repetitive two‐photon imaging of microglia in the spinal cord of Cx3cr1^GFP^ mice. The approach was successfully applied in a laser ablation model of SCI. Furthermore, Ahanonu et al. have recently described chronic in vivo imaging in the spinal cord of awake mice (Ahanonu et al. 2024). To allow for continual optical access to the spinal cord, the authors designed a three‐part implantable chamber which included a stabilization plate. Fluoropolymer‐based inhibition effectively minimized laminectomy‐associated fibrosis, which impedes imaging clarity. Finally, to overcome motion in freely moving mice, the authors developed a multistep motion correction workflow. Once the chamber was implanted, subsequent attachment to the imaging apparatus took less than 1 min and did not require the use of anesthesia. Ahanonu et al. reported long‐term optical clarity for up to 557 days. Animal behavior and spinal cord integrity were preserved, and microgliosis was only observed transiently post‐surgery. This imaging approach was applied to study microglia in a neuropathic pain model (spared nerve injury), using Cx3cr1^eGFP^ mice. Figure 1 summarizes the approaches which can be used to label and study murine microglia in preclinical SCI.

**FIGURE 1:**
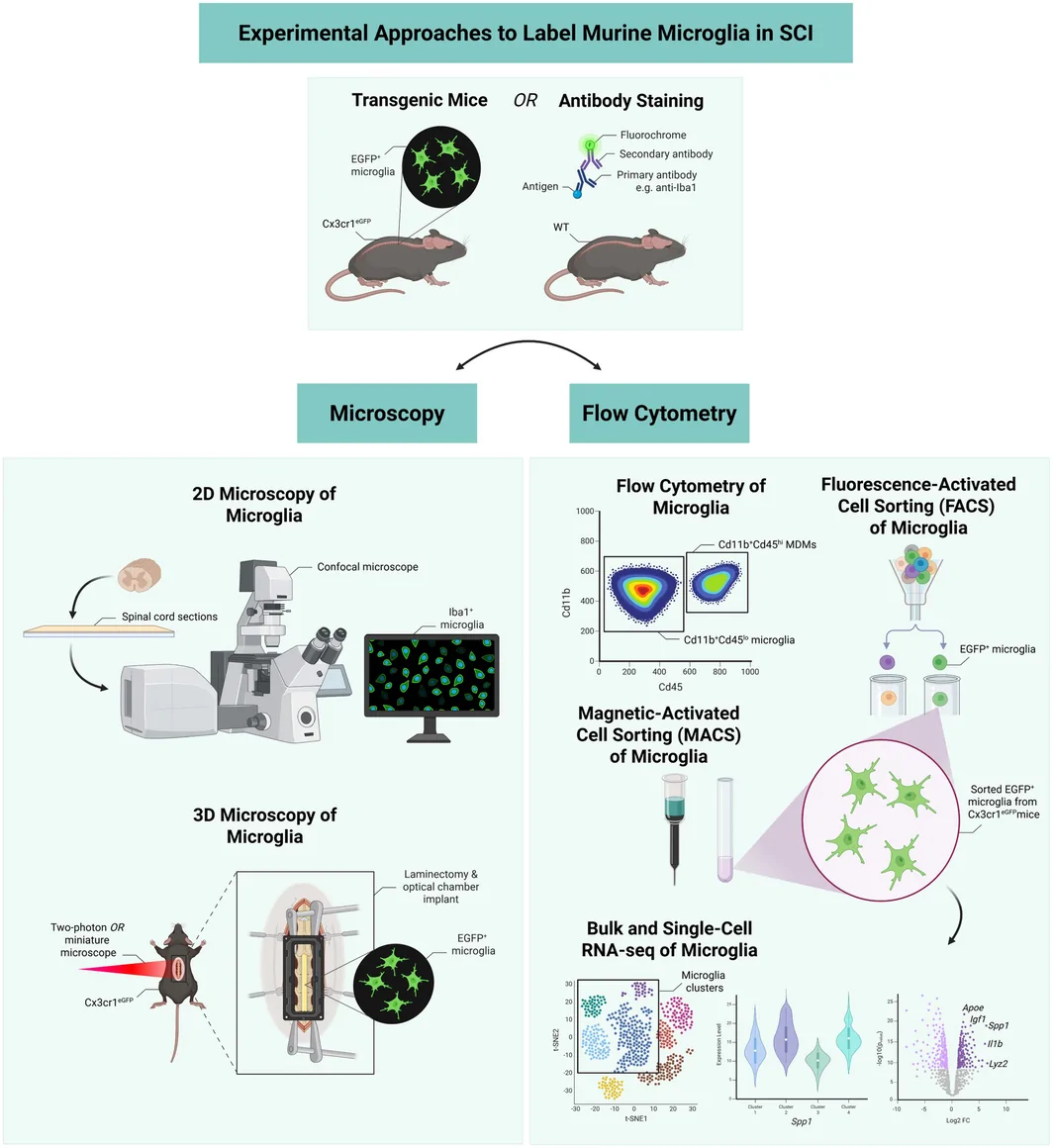
Schematic of experimental approaches discussed in Section 2, which are available to label murine microglia for study in preclinical SCI. Microglia can be labeled with antibodies (e.g., anti‐Iba1), or through genetic manipulation (e.g., Cx3cr1^eGFP^). These labeled microglia can be studied using imaging approaches (2D or 3D microscopy) or flow cytometry‐based approaches (flow cytometry or cell sorting for transcriptomics). Created using BioRender.

## 3 Microglial Markers and Tools in the Context of SCI

### 3.1 The Transcriptional Profile of Microglia After SCI

As microglia undergo transcriptional (among other) changes in a trauma microenvironment such as SCI (Figure 2), it is important to consider the (i) time (in relation to injury), (ii) efficacy (in the murine injury model), and (iii) specificity (to microglia) when selecting an appropriate marker to study these cells. Here we will evaluate the current antibody markers and genetic tools which have been developed (or repurposed) in order to visualize or manipulate microglia, in the context of SCI in mouse. This section is summarized in Table 2. RNA‐seq studies of microglia in SCI consistently show that the homeostatic cluster(s) dominating the uninjured spinal cord are replaced by injury‐associated cluster(s) after SCI, and these are characterized by significant changes in microglial gene expression. Although the differential expression varies across the temporal phases of SCI, studies report many of the same genes to be upregulated in the acute (Hakim et al. 2021; Hamel et al. 2023; Li, Wu, et al. 2022; Milich et al. 2021), subacute (Brennan et al. 2022; Fisher et al. 2022; Hakim et al. 2021; Hamel et al. 2023; Li, Wu, et al. 2022; Milich et al. 2021; Noristani, Gerber, et al. 2017; Wahane et al. 2021; Wang et al. 2022; Yao et al. 2022) and sub‐chronic/chronic (Brennan et al. 2022; Fisher et al. 2022; Hakim et al. 2021; Hamel et al. 2023; Li, Wu, et al. 2022; Wahane et al. 2021; Yao et al. 2022) phases. Moreover, there is a significant overlap between upregulated genes in microglia post‐SCI and DAM (Fisher et al. 2022; Wahane et al. 2021), a microglial phenotype described in a model of Alzheimer's disease (Keren‐Shaul et al. 2017), suggesting that a subset of SCI‐induced genes are upregulated in reactive microglia in general. Microglia post‐SCI express genes including *Abca1, Adam8, Apoe, Axl, Cd63, Cd9, Csf1, Ctsb, Fabp5, Fth1, Fn1, Gfap, Gpnmb, Ifitm3, Igf1, Il1b, Itgax, Lgals3, Lgals3bp, Lpl, Lyz2, Mmp12, Ms4a7, Msr1, Pf4, Spp1, Stat1, Tlr2, Tubb5*, and *Vim* (Brennan et al. 2022; Fisher et al. 2022; Hakim et al. 2021; Hamel et al. 2023; Li, Wu, et al. 2022; Milich et al. 2021; Noristani, Gerber, et al. 2017; Wahane et al. 2021; Wang et al. 2022; Yao et al. 2022); however, these are not microglial‐specific and some are in fact typically used as markers for other cell types, such as *Gfap* (astrocyte marker) and *Itgax* (dendritic cell marker). As such, these markers should be used in conjunction with an established microglial marker, such as *Iba1*. While these studies have shown that some microglial markers are also upregulated (e.g., *C1qa, Clec7a, Cst7, Ctsd, S100a8, Trem2*) or at least stably‐expressed (e.g., *Cd45 (Ptprc), Cd68, F4/80 (Adgre1), Iba1 (Aif1)*) post‐trauma, it is important to note that a subset of homeostatic microglial markers are downregulated after SCI; these include *Cx3cr1, P2ry12, P2ry13, Selplg, Siglech, Tgfbr1*, and *Tmem119*. Beyond these temporal shifts in gene expression (Hakim et al. 2021), microglia remain functionally active well into the chronic phase of SCI, contributing to the long‐term maintenance of the glial scar border (Bellver‐Landete et al. 2019) and of chronic pain (Hains and Waxman 2006). As marker efficacy and specificity can differ by injury phase, it should not be assumed that a tool validated in the acute/subacute window remains equally reliable at sub‐chronic/chronic timepoints, and this represents an important gap for future tool validation.

**FIGURE 2:**
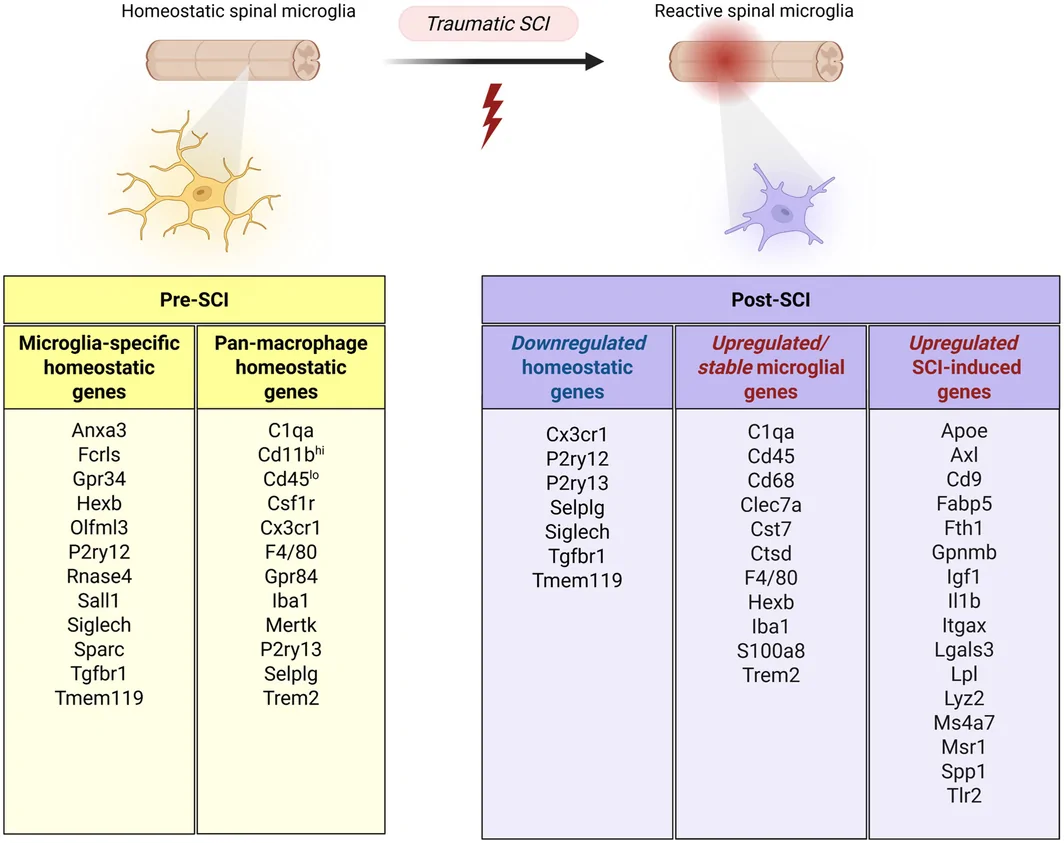
Homeostatic microglia markers can be divided into microglia‐specific and pan‐macrophage genes. These markers may remain stable or become downregulated after SCI. Another subset of genes will be specifically upregulated after SCI, although these are not microglia‐specific. Created using BioRender.

**TABLE 2:** Summary of markers with tools available to label microglia in the mouse, as evaluated in Section 3.

Gene	Specificity	Expression in SCI	IHC antibody	Transgenic line(s)	Application of transgenic lines in models of CNS injury/disease
Aif1 (Iba1)	Pan‐macrophage	Stable expression	Yes (Imai et al. 1996; Ito et al. 1998)	Iba1^EGFP^ (Hirasawa et al. 2005)	Iba1^EGFP^ reported in laser‐induced brain injury (Hefendehl et al. 2014)
Csf1r (c‐fms)	Pan‐macrophage	Un‐determined	Yes (Sasmono et al. 2003)	Csf1r^EGFP^ “MacGreen” (Erblich et al. 2011; Sasmono et al. 2003) ΔCsf1r^ECFP^ “MacBlue” (Ovchinnikov et al. 2008; Sauter et al. 2014) Csf1r^mApple^ (Hawley et al. 2018) Csf1r^FRed^ (Grabert et al. 2020)	MacGreen reported in: Choroidal neovascularization (Crespo‐Garcia et al. 2015) MCAO (Chen et al. 2015) Prion disease (Gómez‐Nicola et al. 2013) Retinopathy (Brockmann et al. 2018)
Cx3cr1	Pan‐macrophage	Down‐regulated	Yes (Hughes et al. 2002)	Cx3cr1^GFP^ (Jung et al. 2000) Cx3cr1^Cre^ (Goldmann et al. 2013; Yona et al. 2013) Cx3cr1^CreER^ (Goldmann et al. 2013; Yona et al. 2013) Cx3cr1^GFP^Ccr2^RFP^ (Mizutani et al. 2012; Saederup et al. 2010) Cx3cr1^ccre^Sall1^ncre^ (Kim et al. 2021)	Cx3cr1^GFP^ and/or Cx3cr1^Cre^/ Cx3cr1^CreER^ reported in: SCI (Hakim et al. 2021; Hamel et al. 2023; Li et al. 2020; Noristani, Gerber, et al. 2017; Wahane et al. 2021) And various other models including: Alzheimer's disease (Steffen et al. 2018) EAE (Goldmann et al. 2013) Parkinson's disease (Virgone‐Carlotta et al. 2013) TBI (Alves et al. 2025) Cx3cr1^GFP^Ccr2^RFP^ reported in: SCI (Dooley, Lemmens, Vangansewinkel, et al. 2016) Cuprizone‐induced demyelination (Vankriekelsvenne et al. 2022) EAE (Mizutani et al. 2012)
Fcrl2 (Fcrls)	Microglia‐specific	Un‐determined	Yes^a^	Fcrls^Cre^ (Kaiser et al. 2024)	Fcrls^Cre^ reported in healthy tissue only
Hexb	Microglia‐specific	Stable expression	Yes^a^	Hexb^tdTomato^ (Masuda et al. 2020) Hexb^CreERT2^ (Masuda et al. 2020)	Hexb^tdTomato^ reported in: SCI (Walsh, Colbert, et al. 2025) Alzheimer's disease, ALS, Cuprizone‐induced demyelination, Huntington's disease (Frosch et al. 2025) EAE, Facial nerve axotomy (Masuda et al. 2020) TLE (Villalba‐Benito et al. 2026)
P2ry12	Microglia‐specific	Down‐regulated	Yes (Haynes et al. 2006)	P2ry12^CreER^ (McKinsey et al. 2020)	P2ry12^CreER^ reported in EAE, MCAO (McKinsey et al. 2020)
Sall1	Microglia‐specific	Un‐determined	Yes^a^	Sall1^GFP^ (Buttgereit et al. 2016; Takasato et al. 2004) Sall1^CreER^ (Buttgereit et al. 2016; Inoue et al. 2010) Cx3cr1^ccre^Sall1^ncre^ (Kim et al. 2021)	Sall1^GFP^ reported in EAE (Buttgereit et al. 2016; Mrdjen et al. 2018)
Tmem119	Microglia‐specific	Down‐regulated	Yes (Bennett et al. 2016)	Tmem119^EGFP^ (Kaiser and Feng 2019) Tmem119^CreERT2^ (Kaiser and Feng 2019) Tmem119^tdTomato^ (Ruan et al. 2020)	Tmem119^EGFP^ reported in: Brain metastasis (Ma et al. 2024) Intracerebral hemorrhage (Ye et al. 2022) Tmem119^CreER^ reported in: SCI (Zeng et al. 2026) MCAO (Del Águila et al. 2024)

Abbreviations: ALS, amyotrophic lateral sclerosis; EAE, experimental autoimmune encephalomyelitis; MCAO, middle cerebral artery occlusion; TBI, traumatic brain injury; TLE, temporal lobe epilepsy.

^a^ Antibodies commercially available but use in murine CNS tissue has not been published.

### 3.2 Aif1/Iba1: Anti‐Iba1, Iba1^EGFP^

*Iba1* (ionized calcium‐binding adapter molecule 1/*Aif1*, allograft inflammatory factor 1) is the prototypical marker used for IHC visualization of microglia. However, it is also expressed by MDMs and thus is not microglia‐specific (Butovsky et al. 2014; Grassivaro et al. 2020; Imai et al. 1996) in the context of peripheral infiltration. Furthermore, while in the CNS parenchyma anti‐Iba1 labeling is restricted to microglia, it was also reported to label perivascular, choroid plexus, and meningeal macrophages in the rodent brain (Ito et al. 1998). A major advantage of *Iba1* is that it is also expressed by reactive microglia. It has been reported to be stably expressed in preclinical SCI (Hamel et al. 2023) and upregulated in several models of CNS injury/disease (Ito et al. 1998; Vankriekelsvenne et al. 2022). Although many SCI studies do not comment on the stability of *Iba1* expression, its exclusion from lists of downregulated genes and its use in IHC as a microglial marker in tissue post‐injury (Dooley, Lemmens, Ponsaerts, and Hendrix 2016; Hakim et al. 2021; Noristani, Gerber, et al. 2017; Wahane et al. 2021; Walsh, Colbert, et al. 2025) would further indicate its stability. Although most commonly used as an antibody marker, a reporter line targeting the *Iba1* promoter was developed to allow for in vivo labeling of microglia: Iba1^EGFP^ (Hirasawa et al. 2005). IHC analyses showed that all enhanced green fluorescent protein (EGFP)^+^ cells had ramified morphology and were Iba1^+^. Iba1^EGFP^ has been independently adapted and re‐validated in rat (VanRyzin et al. 2021). Thus, *Iba1* will robustly label microglia in the lesion post‐SCI but will also label infiltrating MDMs as well as CAMs.

### 3.3 Csf1r: Anti‐Csf1r/Anti‐Cd115, Csf1r^EGFP^ , ΔCsf1r^ECFP^ , Csf1r^mApple^ , and Csf1r^FRed^

*Csf1r* (macrophage colony‐stimulating factor 1 receptor) is a cytokine receptor universally expressed by cells of the mononuclear phagocyte system (MPS), which includes microglia as well as committed progenitors, monocytes, tissue‐resident macrophages, and classical dendritic cells (Grabert et al. 2020). The *Csf1r* promoter has been targeted to develop several transgenic reporter mice, all of which label microglia. Although anti‐Csf1r (anti‐Cd115) antibodies are available and are used in IHC and flow cytometry, the membrane expression of this receptor is unreliable as it is subject to endocytosis and degradation upon ligand binding (Hawley et al. 2018). Furthermore, *Csf1r* expression in SCI is unclear, having been reported to be stable (Wahane et al. 2021), downregulated (Fisher et al. 2022) and upregulated (Hamel et al. 2023) post‐injury, depending on the study; although it has been shown to be relatively stable in several other models of CNS injury/disease (Masuda et al. 2020). Moreover, the reporter lines discussed here have not yet been tested in a SCI environment. The *Csf1r* promoter was first targeted in the development of a “MacGreen” reporter: Csf1r^EGFP^ (Sasmono et al. 2003). EGFP expression was shown to be consistent with F4/80 expression, and in the brain it was restricted to microglia and CAMs. In the periphery, MDMs as well as blood monocytes were labeled. MacGreen mice have high efficiency of labeling microglia, with 100% of EGFP^+^ cells being Csf1r^+^ and 100% of Iba1^+^F4/80^+^ cells being EGFP^+^ (Erblich et al. 2011). This tool has been cross‐validated in a nontraumatic model of CNS injury (Chen et al. 2015). The “MacBlue” reporter, ΔCsf1r^ECFP^, contains a modified *Csf1r* promoter which indirectly drives expression of enhanced cyan fluorescent protein (ECFP) (Ovchinnikov et al. 2008). The modification removes the reporter expression in several MPS populations (Sauter et al. 2014), thus, this tool targets a subset of the MPS, namely: monocytes, most classical dendritic cells, and select tissue macrophage populations including microglia. Efficiency of labeling in Cd45^lo^Cd11b^+^ microglia is, however, superior in MacGreen mice.

Csf1r^mApple^ was developed under the same promoter as MacGreen, and mApple expression is consistent with EGFP expression in Csf1r^EGFP^ mice (Hawley et al. 2018). Development of Csf1r^mApple^ was driven by a need for reporters compatible with existing transgenic tools, many of which are EGFP‐based. To demonstrate the feasibility of crossing this novel reporter line, Csf1r^mApple^ was crossed with ΔCsf1r^ECFP^ (Csf1r^mApple^ΔCsf1r^ECFP^), which allowed for distinguishing subpopulations of the MPS. The most recently developed reporter line utilizing the *Csf1r* promoter expresses Fusion Red (FRed): Csf1r^FRed^ (Grabert et al. 2020). In brain tissue, FRed is expressed in Cd45^lo^Cd11b^hi^ microglia and Cd45^hi^Cd11b^lo^ CAMs. FRed is also expressed by MDMs in all other organs and blood monocytes, among other MPS populations. Flow cytometry revealed that FRed aligned with expression of Cd115 (Csf1r). Grabert et al. emphasize the opportunity to insert a desired expression cassette (such as *Cre*) into the *Csf1r* promoter for future studies targeting the MPS (Grabert et al. 2020). Overall, the efficacy of using *Csf1r*‐based tools for labeling microglia in SCI is undetermined. While there are several *Csf1r*‐based tools to choose from, all will label at least some monocyte/macrophage populations as well as microglia.

### 3.4 Cx3cr1: Anti‐Cx3cr1, Cx3cr1^GFP^ , Cx3cr1^Cre^ , and Cx3cr1^CreER^

*Cx3cr1* (fractalkine receptor) is a chemokine receptor highly, although not exclusively, expressed by microglia (Butovsky et al. 2014; Gautier et al. 2012; Milich et al. 2021) and its promoter is the most common marker used for genetic targeting of microglia. In the CNS, *Cx3cr1* is specifically expressed by microglia and CAMs; however, the fractalkine receptor is expressed by other immune cells in the periphery, including monocytes, MDMs, and dendritic cells. The generation of Cx3cr1^GFP^ reporter mice (Jung et al. 2000) marked the first time that microglia could be studied in vivo, and this paper has been cited extensively across the field. In the CNS, microglia were the only glial cell to be GFP^+^. MDMs in the periphery were GFP^−^. However, blood monocytes and subsets of natural killer cells and dendritic cells were labeled. The knock‐out approach in the generation of Cx3cr1^GFP^ also meant that reporter mice must be bred as heterozygotes to preserve endogenous expression of one allele of *Cx3cr1*.

Cx3cr1^Cre^ and Cx3cr1^CreER^ were also generated (Yona et al. 2013) to allow for constitutive and conditional expression of *Cre* recombinase in Cx3cr1^+^ cells, respectively, and the latter can be used to distinguish microglia from MDMs based on the high turnover of MDMs. Both *Cre* tools were crossed with Rosa26 (R26)‐yfp reporters (Cx3cr1^Cre^R26^yfp^; Cx3cr1^CreER^R26^yfp^), and yellow fluorescent protein (YFP) expression was compared to GFP expression in Cx3cr1^GFP^ reporter mice (Jung et al. 2000). Microglia were found to be GFP^+^YFP^+^ and the majority of MDMs were GFP^−^YFP^+^. The newly‐generated Cx3cr1^Cre^ and Cx3cr1^CreER^ were shown to have high recombination as well as high specificity to microglia in the CNS (Goldmann et al. 2013). Up to 99% of Cd45^lo^Cd11b^+^ microglia and Iba1^+^ microglia expressed YFP without ectopic expression in Iba1^−^ cells. YFP was, however, expressed in blood monocytes. Cx3cr1^Cre^ mice were specifically used to study microglia in experimental autoimmune encephalomyelitis (EAE). To distinguish microglial effects in EAE from that of MDMs, Goldmann et al. exploited the differential turnover rates of these two immune cell populations. Tamoxifen was administered several weeks prior to EAE induction, thus infiltrating MDMs were not targeted as these cells are short‐lived and were replaced in the circulation by Cx3cr1^−^ monocytes prior to infiltration. YFP^+^Cd11b^+^ microglia could be distinguished from YFP^−^Ly‐6C^hi/lo^ infiltrating monocytes. In Cx3cr1^CreER^ mice, cells must express *Cx3cr1* at the time of tamoxifen induction and be self‐renewing in order to be targeted. Parkhurst et al. have similarly generated Cx3cr1^CreER^ mice for the study of microglia (Parkhurst et al. 2013). These mice conditionally express EYFP under the promoter, with > 98% of EYFP^+^ cells in brain tissue being Iba1^+^ microglia. Crossing to R26‐DsRed reporter mice (Cx3cr1^CreER/+^R26^DsRed/+^), tamoxifen induction showed highly efficient recombination in microglia, which could be specifically targeted if tamoxifen was given 30 days prior to experimental induction; almost all microglia remained dsRed^+^ while only a minority of peripheral Cd11b^+^ cells were labeled.

Anti‐Cx3cr1 antibodies are also available to label microglia in situ and in the healthy CNS and were shown to label resident microglia as well as CAMs (Hughes et al. 2002). In this study, *Cx3cr1* expression was either upregulated or stable in models of CNS pathology. However, sequencing studies have generally reported *Cx3cr1* to be downregulated in preclinical SCI (Fisher et al. 2022) and other models of CNS injury/disease (Hammond et al. 2019; Keren‐Shaul et al. 2017; Masuda et al. 2020; Mrdjen et al. 2018). Despite this, Cx3cr1^GFP/+^ and Cx3cr1^CreERT2^R26^tdT^ have successfully been used to isolate microglia for sequencing in a number of studies, including SCI models (Hamel et al. 2023; Li et al. 2020; Noristani, Gerber, et al. 2017; Wahane et al. 2021). In summary, *Cx3cr1*‐based tools remain a popular choice for studying microglia, despite shared expression with MDMs.

### 3.5 Fcrls: Fcrls^Cre^

*Fcrls* (Fc receptor‐like S, scavenger receptor/*Fcrl2*) is a microglia‐specific gene (Butovsky et al. 2014; Gautier et al. 2012) with undetermined function. While anti‐Fcrls antibodies have not been applied to IHC studies of microglia, a novel *Cre* line using the *Fcrls* promoter has recently been developed: Fcrls^Cre^ (Kaiser et al. 2024). Fcrls^Cre^ constitutively targets microglia, avoiding the requirement of tamoxifen induction as is the case in *CreERT2* lines. Flow cytometry of Fcrls^Cre^ showed virtually no *Cre* activity in Cd45^+^ leukocytes, including monocytes. Kaiser et al. have crossed this *Cre* line with four reporter lines (including R26^yfp^ and Ai14^tdT^) and demonstrated, regardless of the reporter allele, highly efficient and specific targeting of microglia in CNS tissue (Kaiser et al. 2024). There was near‐complete labeling of Cd11b^+^Cd45^lo^ microglia and similarly, IHC revealed almost all parenchymal Iba^+^ microglia were targeted while almost all reporter‐labeled cells were Iba1^+^. CAMs have been reported to also express *Fcrls* (Hamel et al. 2023; Jordão et al. 2019) and were efficiently targeted in this *Cre* line. There was also modest labeling in spleen and liver macrophages which may represent subsets of these tissue‐resident macrophages. An advantage to this *Cre* line is that the knock‐in approach retained *Fcrls* mRNA expression in the transgenic mice. Overall, Fcrls^Cre^ has robust targeting of microglia, although it also targets CAMs. A notable limitation is that its effectiveness for labeling SCI is undetermined. While one study reported the stability of *Fcrls* expression in a model of demyelination (Hammond et al. 2019), it has also been reported to be downregulated in SCI (Hamel et al. 2023) and several other models of CNS injury/disease (Frosch et al. 2025; Masuda et al. 2020).

### 3.6 Hexb: Hexb^tdTomato^ , Hexb^CreERT2^

*Hexb* (beta‐hexosaminidase subunit beta) was unexpectedly found by Hickman et al. to be the highest expressing transcript among genes unique to microglia (Hickman et al. 2013). Similarly, Butovsky et al. identified *Hexb* as a microglial‐specific gene when describing the microglial signature, with no expression in Ly6C^+^ monocytes, tissue‐specific MDMs or other CNS‐resident cells (Butovsky et al. 2014). *Hexb* has been reported to be stably expressed in preclinical SCI (Hamel et al. 2023; Wahane et al. 2021) as well as in the DAM phenotype (Keren‐Shaul et al. 2017). Although one study reported that MDMs and CAMs had increased expression of *Hexb* in the sub‐chronic phase of SCI (Hamel et al. 2023), both Masuda et al. and Frosch et al. have shown stable and specific expression of *Hexb* in microglia across seven models of CNS pathology (Frosch et al. 2025; Masuda et al. 2020), including EAE which involves influx of peripheral immune cells as occurs after SCI. To this end, Masuda et al. have used the *Hexb* promoter to develop two transgenic tools: Hexb^tdTomato^ and Hexb^CreERT2^ mice, to visualize and target microglia, respectively. Hexb^tdTomato^ was shown to have high efficacy and specificity for labeling microglia, including in spinal cord tissue; virtually all Iba1^+^ cells were double‐positive for tdTomato (tdT). In contrast, only very low detection of tdT was found in CAMs (much lower than in Cx3cr1^GFP/+^ mice). In the periphery, tdT was detected in a subset of kidney macrophages only. Hexb^tdTomato^ has been re‐validated (although not independently) and further has been cross‐bred with Cx3cr1^GFP^ and Thy1^GFP^, showing no overlap of tdT expression with CAMs or neurons, respectively (Frosch et al. 2025). Hexb^tdTomato^ mice have been effectively used to label microglia in preclinical SCI (Walsh, Colbert, et al. 2025). While homozygous (Hexb^tdT/tdT^) microglia show reduced *Hexb* mRNA expression (Masuda et al. 2020) and Hexb^CreERT2/CreERT2^ showed reduced Hexb enzyme activity (Faust et al. 2023), no effect on cellular phenotype was found. Similarly, Hexb^CreERT2^ mice efficiently target microglia, with very minimal targeting of CAMs. High and specific recombination was shown through FACS and histological analysis, crossing Hexb^CreERT2^ with R26‐yfp reporter mice (Hexb^CreERT2/CreERT2^R26^yfp/yfp^). Hexb^CreERT2^ was also crossed with Csf1r‐deficient mice (Hexb^CreERT2/CreERT2^Csf1r^fl/fl^) whereby tamoxifen induction led to a significant decrease in microglial numbers without affecting CAM numbers. Since *Csf1r* expression is required for both microglial and CAM survival, this demonstrated selective targeting of microglia using the *Cre*‐dependent *Hexb* promoter. Thus, *Hexb* is a suitable marker for labeling and targeting microglia in SCI, due to its stable expression and cell specificity. Use of anti‐Hexb antibodies for studying microglia has not been published.

### 3.7 P2ry12: Anti‐P2ry12, P2ry12^CreER^

*P2ry12* (P2Y purinoceptor 12) is a marker specific to and highly expressed by microglia (Bédard et al. 2007; Butovsky et al. 2014; Milich et al. 2021; Sasaki et al. 2003). Anti‐P2ry12 antibodies colocalize with EGFP in Cx3cr1^EGFP^ tissue and with anti‐Cd11b (Haynes et al. 2006). However, immunoreactivity was concurrently shown to be downregulated in activated microglia. *P2ry12* expression has consistently been reported to be downregulated in preclinical SCI (Hamel et al. 2023; Milich et al. 2021; Wahane et al. 2021) as well as in various other models of CNS injury/disease characterized by microglial activation (Bennett et al. 2016; Hammond et al. 2019; Jordão et al. 2019; Keren‐Shaul et al. 2017; Masuda et al. 2020), often emerging as one of the top downregulated genes. The *P2ry12* promoter has been targeted to generate a *Cre*‐driven genetic tool to study microglia: P2ry12^CreER^ (McKinsey et al. 2020). The main advantage of this tool is specificity, which was demonstrated by crossing with Rosa26^Ai14^. P2ry12^CreER^Rosa26^Ai14^ have high recombination efficiency and specificity, including in spinal cord tissue; tdT^+^ expression was high in Cd11b^+^CD45^int^ and Iba1^+^ cells. There was some labeling in a subset of choroid plexus and meningeal (but not perivascular) macrophages as well. Furthermore, while there was moderate recombination observed in spleen macrophages, virtually no circulating lymphocytes or monocytes were tdT^+^. Another advantage of this tool is that the knock‐in approach preserves *P2ry12* expression, and while homozygous mice show reduced *P2ry12* transcripts, no effects on microglial phenotype were detected (McKinsey et al. 2020), and no change in protein levels was found (Faust et al. 2023). To address the downregulation of *P2ry12* expression in injury/disease, McKinsey et al. show that pretreatment with tamoxifen 7 days prior to induction of ischemia (middle cerebral artery occlusion) or neuroinflammation (EAE) allows identification of microglia through tdT expression, despite lack of *P2ry12* immunoreactivity. Furthermore, in P2ry12^CreER^Rosa26^Ai14^Cx3cr1^EGFP^ mice with EAE, P2ry12^CreER^ was shown to target microglia (tdT^+^EGFP^+^) but not infiltrating monocytes (tdT^−^EGFP^+^). However, the authors acknowledge that the recombination efficiency would likely be reduced in this instance, which would present a limitation. Overall, this tool offers potential for labeling microglia with high selectivity after SCI.

### 3.8 Sall1: Sall1^GFP^ , Sall1^CreER^

*Sall1* (Sal‐like protein 1) is a microglia‐specific gene (Butovsky et al. 2014; Gautier et al. 2012) that regulates the transcriptional signature of microglia (Buttgereit et al. 2016). Sall1^GFP^ (Takasato et al. 2004) and Sall1^CreER^ (Inoue et al. 2010) mice, both knock‐in tools, were originally developed to target mesenchymal cells in the developing kidney (which are also Sall1^+^). However, Buttgereit et al. have repurposed these as tools for labeling and targeting microglia, respectively (Buttgereit et al. 2016). Sall^GFP/+^ reporter mice showed *Sall* expression which was largely restricted to microglia in the CNS. IHC analyses revealed colocalization of GFP^+^ and Iba1^+^ cells, with Iba1^−^ cells almost exclusively GFP^−^. Flow analyses showed all Cd11b^+^Cd45^lo^ microglia were GFP^+^; Cd45^+^ cells outside the CNS were GFP^−^. GFP^+^Cd45^−^ cells in the CNS were scarce and approximately 5% of CAMs were GFP^+^. In another study, Sall1^GFP/+^ was shown to label microglia but not CAMs or dendritic cells (Mrdjen et al. 2018). In the EAE model of neuroinflammation, microglia remain Sall1^+^ and notably, infiltrating immune cells are Sall^−^ (Buttgereit et al. 2016). Sall1^CreER^ have successfully been used for gene manipulation in microglia, where Sall1^CreER^Csf1r^fl/fl^ mice efficiently deplete microglia (Buttgereit et al. 2016). Moreover, crossing Sall1^CreER^ with R26‐YFP reporter mice (Sall1^CreER^R26^yfp^) showed high (> 90%) recombination efficiency and specificity to microglia. However, while Sall1^CreER^ does not label CAMs, ectopic expression in Cd45^−^Cd11b^−^ cells in the CNS has been reported (Masuda et al. 2020). *Sall1* expression has not been reportedly downregulated in studies of preclinical SCI (Hamel et al. 2023; Milich et al. 2021; Wahane et al. 2021) or in DAM (Keren‐Shaul et al. 2017), however, Jordão et al. reported its downregulation in EAE (Jordão et al. 2019) while Masuda et al. and Frosch et al. have shown low expression of *Sall1* in several other models of CNS pathology (Frosch et al. 2025; Masuda et al. 2020). Anti‐Sall1 antibodies are commercially available but have not been used to study microglia as yet. As such, while there are no reports on the efficacy of *Sall1*‐based tools to study microglia in SCI, if stable, these would offer greater selectivity than pan‐macrophage markers.

### 3.9 Tmem119: Anti‐Tmem119, Tmem119^EGFP^ , Tmem119^CreERT2^ , and Tmem119^tdTomato^

*Tmem119* (Transmembrane protein 119) is similarly a microglial‐specific gene (Butovsky et al. 2014; Gautier et al. 2012), highly and selectively expressed by parenchymal microglia; in situ hybridization showed neither CAMs nor peripheral CD45^+^ immune cells express this marker (Bennett et al. 2016). Anti‐Tmem119 was developed and tested in several disease models including central (optic nerve crush) injury (Bennett et al. 2016), which as a model of traumatic CNS injury would have parallels with SCI. All Iba1^+^ parenchymal cells were double positive for *Tmem119* with the same intensity as in healthy tissue. Furthermore, in Ccr2^RFP/+^ mice with optic nerve crush, where infiltrating monocytes express red fluorescent protein (RFP), most Iba1^+^ cells were Tmem119^+^RFP^−^, while the few Iba1^+^Tmem119^−^ cells were RFP^+^.

Tmem119^EGFP^ and Tmem^CreERT2^ have been developed as tools with high efficacy and specificity for labeling parenchymal microglia (Kaiser and Feng 2019). In Tmem119^EGFP^, almost all EGFP^+^ cells were also Iba1^+^Tmem119^+^ in the brain parenchyma and similarly, all CD11b^+^CD45^lo^ cells were EGFP^+^. CAMs as well as CD45^+^ monocytes were EGFP^−^. Similarly, Tmem119^CreERT2^ crossed with Ai14 reporter mice (Tmem119^CreERT2/+^Ai14^+/−^) had high recombination and specificity. In the brain parenchyma, almost all Iba1^+^ cells were tdT^+^ and the converse was true. A small proportion of CD11b^+^CD45^hi^ CAMs and a very small percentage of peripheral CD45^+^ immune cells were also tdT^+^. By virtue of the knock‐in design, endogenous expression of *Tmem119* is preserved in these transgenic mice, although reduced protein levels have been noted (Faust et al. 2023). In contrast, Vankriekelsvenne et al. tested the validity of *Tmem119* in four in vivo models of CNS injury/disease, including EAE, where tissue from inflammatory lesions in the spinal cord was used (Vankriekelsvenne et al. 2022). In all models, *Tmem119* expression was significantly reduced. The authors note that their models correlate with early microglial activation and as such, *Tmem119* may be unsuitable as a marker for at least the acute and subacute stages of SCI. Ectopic expression of this gene was identified but only in peripheral cells which are of irrelevance to SCI. The authors conclude that *Tmem119* is a reliable microglial marker for homeostatic microglia in the healthy CNS. This homeostatic gene has been reported to be highly downregulated in preclinical SCI (Hakim et al. 2021; Hamel et al. 2023) and indeed other models of CNS injury/disease (Frosch et al. 2025; Hammond et al. 2019; Jordão et al. 2019; Keren‐Shaul et al. 2017; Masuda et al. 2020; Masuda et al. 2019). However, one study has effectively utilized Tmem119^CreER^ mice to target microglia in acute to sub‐chronic SCI (Zeng et al. 2026). Knock‐in Tmem119^tdTomato^ reporter mice have also been generated, where tdT expression is co‐expressed with anti‐Tmem119 and colocalized with Iba1^+^ cells (Ruan et al. 2020). A small proportion of choroid plexus macrophages were also tdT^+^ but there was no expression in perivascular or meningeal macrophages. Flow cytometry revealed almost all Cd11b^+^Cd45^lo^ microglia were tdT^+^ while a small number of infiltrating Cd11b^+^Cd45^hi^ cells were also tdT^+^. Overall, *Tmem119*‐based tools have high specificity for microglia without targeting monocytes, however, may be unstable for studying microglia in acute and subacute SCI.

### 3.10 Binary Tools: Cx3cr1^GFP^Ccr2^RFP^ , Cx3cr1^ccre^Sall1^ncre^

Several binary *Cre* systems have been developed to address the lack of specificity of microglial tools. Mizutani et al. propose a combination of *Cx3cr1* and *Ccr2* as markers to distinguish CNS‐resident microglia (in a homeostatic or reactive state) from infiltrating MDMs (Mizutani et al. 2012), using Cx3cr1^GFP/+^Ccr2^RFP/+^ (Saederup et al. 2010). In the healthy CNS, GFP was colocalized with Iba1^+^ microglia; RFP expression was absent. In EAE, GFP expression remained colocalized with Iba1 expression; RFP expression emerged but was restricted to Cd45^hi^ monocytes only and not Cd45^lo^ microglia. Thus, Cx3cr1/Ccr2 double reporter mice can be used as a tool to discriminate microglia from monocyte/macrophage populations in the inflamed CNS. Microglia (Cx3cr1^+^Ccr2^−^) will fluoresce green, while MDMs (Cx3cr1^+^Ccr2^+^) will fluoresce green and red. Cx3cr1^+^Ccr2^+^ has been used to study microglia in preclinical SCI (Dooley, Lemmens, Vangansewinkel, et al. 2016), however, the authors acknowledged that the previously reported downregulation of RFP in CCR2^RFP/+^ (Yamasaki et al. 2014) is a limitation of this model. Cx3cr1^ccre^Sall1^ncre^ have also been generated to specifically target parenchymal microglia (Kim et al. 2021). In Cx3cr1^ccre^Sall1^ncre^R26^tdT^ mice, tdT^+^ cells were shown to be Cd11b^+^Cd45^int^ microglia. Recombination efficiency was, however, only moderate (approximately 25%) in heterozygous animals; likely compromised by the low efficiency of the *Sall1* promoter. tdT^+^ were double positive for *Iba1* and located in the brain parenchyma only, with no expression in CAMs, blood, nor peripheral organs. A RiboTag approach was used to characterize the translatome of the recombined cells which expressed typical microglial genes including *Aif1, Csf1r, Hexb*, and *Tmem119* as well as *Cx3cr1* and *Sall1*. At the time of publication (2021), Kim et al. state Cx3cr1^ccre^Sall1^ncre^ as the only model to exclusively target microglia and promote the exploration of alternative combinations for future research. Thus, binary *Cre* systems offer microglial targeting with greater selectivity; however, the choice of promoters will affect labeling efficiency.

The tools discussed here are a nonexhaustive list of antibodies and reporter/*Cre* lines available to study microglia in SCI. There are other antibodies available which are microglia‐specific [e.g., anti‐Anxa3, anti‐Siglech (Konishi et al. 2017; Zhang et al. 2021)), pan‐macrophage (e.g., anti‐Cd11b, anti‐F4/80 (Perry et al. 1985; Robinson et al. 1986)] and SCI‐induced (e.g., anti‐Apoe, anti‐Igf1; Hakim et al. 2021). Similarly, additional genetic tools that target microglia have been developed and include Cd68^GFP^ (Iqbal et al. 2014), Clec7a^CreERT2^ (Barclay et al. 2024), and Trem2^mKate2^ (Feiten et al. 2026).

## 4 Discussion

The transcriptional profile of microglia, although well characterized, will be significantly altered in the SCI microenvironment. When selecting a tool to study microglia in SCI, the primary factors to prioritize are selectivity in microglia (including expression in myeloid cells and ectopic expression) and stability of expression in SCI models. Other factors to consider include efficiency of recombination (in *Cre* tools), effect on endogenous expression (in all promoter‐driven tools), and commercial availability. The experimental approach chosen to study microglia in a murine model of SCI will also influence the tool or marker of choice. Flow cytometry and cell sorting are well suited to antibody markers (Hakim et al. 2021; Wang et al. 2022); flow cytometry and FACS (but not MACS) can also be used with transgenic lines (Hamel et al. 2023; Noristani, Gerber, et al. 2017; Wahane et al. 2021; Walsh, Colbert, et al. 2025). Similarly, 2D microscopy is compatible with both antibody‐stained and transgenic tissue and IHC can utilize a combination of antibody markers (Dooley, Lemmens, Vangansewinkel, et al. 2016; Hakim et al. 2021; Hamel et al. 2023; Noristani, Gerber, et al. 2017; Wahane et al. 2021; Walsh, Colbert, et al. 2025; Wang et al. 2022). 3D microscopy can facilitate in vivo imaging of microglia in anesthetized or freely moving mice (Evans et al. 2014; Fenrich et al. 2013; Nadrigny et al. 2017; Wu et al. 2022); however, it requires the use of transgenic mice in addition to specific expertise and technologies (e.g., multiphoton microscope) which are less accessible.

The transgenic tools discussed in Section 3 each have strengths and limitations. *Iba1*‐based tools (Hirasawa et al. 2005; Ito et al. 1998) have robust expression in reactive microglia but will label infiltrating MDMs. *Cx3cr1*‐based tools (Goldmann et al. 2013; Hughes et al. 2002; Jung et al. 2000) have very high recombination efficiency but must be bred as heterozygous to avoid *Cx3cr1* knock‐out. CX3CL1/CX3CR1 signaling between neurons and microglia helps maintain microglia in a homeostatic state, and its loss alters microglial recruitment and function in a context‐dependent manner. *Cx3cr1* ^−/−^ mice have shown increased neurotoxicity in several neurodegenerative and neuroinflammatory models of CNS injury/disease (Cardona et al. 2006). By contrast, Donnelly et al. reported improved recovery in *Cx3cr1*‐deficient mice after SCI due to reduced recruitment of cytotoxic MDMs (Donnelly et al. 2011). The knock‐out design of *Cx3cr1*‐based tools means that even “control” animals carry only a single functional *Cx3cr1* allele. This raises the question of whether *Cx3cr1*‐based tools should remain gold standard and underscores the need for microglial labeling strategies that do not interfere with their functional biology. While also targeting MDMs, Cx3cr1^CreER^ can selectively target microglia with tamoxifen pretreatment 4 weeks in advance of SCI induction, although *Cx3cr1* expression is downregulated after SCI. *P2ry12*‐based (Haynes et al. 2006; McKinsey et al. 2020) and *Tmem119*‐based (Bennett et al. 2016; Kaiser and Feng 2019; Ruan et al. 2020; Vankriekelsvenne et al. 2022) tools offer the advantage of being microglia‐specific and preserve endogenous gene expression in their respective promoter‐based tools but these homeostatic genes are among those notably downregulated after SCI. *Csf1r*‐based tools (Erblich et al. 2011; Grabert et al. 2020; Hawley et al. 2018; Ovchinnikov et al. 2008) label all cells of the MPS and thus are not microglia‐specific although expression is relatively stable in reactive microglia. The range of reporters available (EGFP, SCFP, FRed, mApple) offers opportunities for the development of binary tools. *Sall1*‐based tools (Buttgereit et al. 2016) do not label MDMs but have shown ectopic expression in Cd45^−^Cd11b^−^ CNS cells (Masuda et al. 2020). *Fcrls*‐based (Kaiser et al. 2024) and *Hexb*‐based (Masuda et al. 2020) tools are microglia‐specific (although *Fcrls* is also expressed in CAMs, *Hexb* is not) and their *Cre*/reporter lines are knock‐in, preserving endogenous expression of the respective markers. While Hexb^tdTomato^ reporter mice have effectively labeled microglia in injured tissue (Walsh, Colbert, et al. 2025), *Fcrls*‐based tools have not been tested in SCI models so their stability in this context is undetermined. Binary tools, such as Cx3cr1^GFP^Ccr2^RFP^ (Mizutani et al. 2012) and Cx3cr1^ccre^Sall1^ncre^ (Kim et al. 2021) which were considered above, can overcome some of the limitations of single promoter‐based tools and should be considered as a feasible option for studying microglia in SCI. Frosch et al. discuss the limitations of using a single gene marker to distinguish microglia in pathology (Frosch et al. 2025). As such, a multi‐marker approach, like the panel proposed by Mrdjen et al. (2018) will strengthen the specificity of labeling. The inducible nature of *CreERT2* lines can be beneficial to exploit the self‐renewal capacity of microglia, to distinguish long‐lived microglia from short‐lived MDMs in SCI pathology. In relation to stability, the strength of the *Cre* line may be compromised in contexts where the expression of the promoter is affected, as is the case in SCI, where the expression of certain homeostatic genes is downregulated. This is also relevant to transgenic reporter lines and antibodies targeting these microglial markers. Overall, *Cx3cr1*‐based tools are the most heavily cross validated and are typically used as a benchmark against which other tools are compared. Faust et al. do provide a comprehensive comparison of several *Cre* lines: Cx3cr1^CreER^, Hexb^CreER^, P2ry12^CreER^ and Tmem119^CreER^ (Faust et al. 2023); while Chappell‐Maor et al. provide a similar comparison between Cx3cr1^CreER^ and Sall^CreER^ (Chappell‐Maor et al. 2020).

Age and sex are additional variables relevant to microglial marker selection, though a full discussion is beyond this review's scope. The transcriptional profile of microglia is significantly altered in aging, with Hickman et al. reporting one third of microglial transcripts being differentially expressed in aged mice; including half of the genes of the sensome (Hickman et al. 2013). Microglia are also sexually dimorphic whereby differences in male and female microglia have been demonstrated at the levels of the transcriptome, proteome, structure, and function (Guneykaya et al. 2018; Villa et al. 2018). These differences are attributed to a combination of differing hormonal profiles, sex chromosomes, epigenetics, and environmental factors (Han et al. 2021; Stewart et al. 2020). In the context of preclinical SCI, Stewart et al. demonstrated that the inflammatory profile differs depending on both sex and age (Stewart et al. 2021). Taken together, there is a need to consider both variables when choosing microglial markers, particularly for translational and personalized‐medicine applications, given that clinically, males are disproportionately affected by SCI while females are associated with better recovery outcomes (Stewart et al. 2020).

Although this review focuses on studies using murine models of SCI, the markers discussed herein can be effectively applied to alternative murine‐derived systems which have been used to study microglia in preclinical SCI; including murine induced pluripotent stem cell (miPSC)‐derived microglia (Aiyegbusi et al. 2025), 3D CNS organoids (Di Stefano et al. 2024), and ex vivo organotypic spinal cord slices (Walsh, Hill, et al. 2025; Walsh et al. 2023). Important preclinical SCI models such as rat and zebrafish rely on partially distinct labeling strategies for microglia. More broadly, the tools discussed throughout this review have been developed and validated almost exclusively in mice, and the majority have not been validated for use in human tissue. This is a significant translational gap. *Fcrls*, for example, is considered one of the most highly expressed microglia markers but does not have a known human ortholog (Butovsky et al. 2014). A notable exception is Anti‐Tmem119, which was raised against and characterized in both mouse and human (Bennett et al. 2016), demonstrating that cross‐species validation is achievable but has not yet been systematically applied across the field. Until such validation is more widely established, extrapolation of murine microglial labeling strategies to other SCI model species, and indeed, to human or clinical samples, should be made with caution.

Taken together, this review provides a critical evaluation of current tools available for labeling microglia in preclinical SCI in the mouse and their application in standard and emerging experimental approaches. Beyond SCI, a more precise identification of microglial signatures represents an important future direction for patient‐specific therapies, as detailed microglial characterization could enable the identification of different disease stages and guide personalized treatment strategies. In parallel, advances in imaging techniques, including microglia‐specific positron emission tomography (Bonomi et al. 2025), are expected to enable real‐time visualization of microglial signatures in patients (Mirarchi et al. 2024). Finally, the integration of complementary approaches such as spatial transcriptomics, imaging mass cytometry, electron microscopy, and machine learning will allow a more comprehensive characterization of microglia (Paolicelli et al. 2022; Wu et al. 2025).

## Author Contributions

R.C.: conceptualization, writing – original draft, writing – review and editing. J.DS.: conceptualization, writing – review and editing. D.D.: conceptualization, funding acquisition, project administration, resources, supervision, writing – review and editing. All authors gave final approval for publication.

## Funding

This work was supported by University College Dublin and Taighde Éireann—Research Ireland (19/FFP/6642 and 22/FFP‐P/11503 to D. D. and Government of Ireland Postgraduate Scholarship GOIPG/2024/2465 to R. C.).

## Conflicts of Interest

The authors declare no conflicts of interest.

## Data Availability

Data sharing not applicable to this article as no datasets were generated or analysed during the current study.
